# Differential proteomic profile of leukemic CD34+ progenitor cells from chronic myeloid leukemia patients

**DOI:** 10.18632/oncotarget.24938

**Published:** 2018-04-24

**Authors:** Maria Rosaria Ricciardi, Valentina Salvestrini, Roberto Licchetta, Simone Mirabilii, Mattia Forcato, Gabriele Gugliotta, Simona Salati, Fausto Castagnetti, Gianantonio Rosti, Massimo Breccia, Giuliana Alimena, Rossella Manfredini, Silvio Bicciato, Roberto Massimo Lemoli, Agostino Tafuri

**Affiliations:** ^1^ Hematology Unit, Sant'Andrea University Hospital, Department of Clinical and Molecular Medicine, Sapienza University of Rome, Rome, Italy; ^2^ Department of Experimental, Diagnostic and Specialty Medicine, Institute of Hematology “L. and A. Seràgnoli,” University of Bologna, S. Orsola-Malpighi Hospital, Bologna, Italy; ^3^ Department of Biomedical Sciences, University of Modena and Reggio Emilia, Modena, Italy; ^4^ Department of Cellular Biotechnologies and Hematology, Sapienza University of Rome, Rome, Italy; ^5^ Clinic of Hematology, Department of Internal Medicine (DiMI), University of Genoa, Genoa, Italy

**Keywords:** chronic myeloid leukemia, CD34+ cells, proteomic profile, cell signaling, apoptosis

## Abstract

Chronic Myeloid Leukemia (CML) is a stem cell disease sustained by a rare population of quiescent cells which are to some extent resistant to tyrosine kinase inhibitors (TKIs). BCR-ABL oncogene activates multiple cross-talking signal transduction pathways (STP), such as RAS/MEK/ERK, PI3K/Akt, Wnt and STAT5, contributing to abnormal proliferation of clonal cells. From this perspective, the aim of this study was to analyze the expression and activation profile of STP involved in the mechanisms of cell proliferation/quiescence and survival of the progenitor CD34+ cells from chronic phase (CP) CML.

Our results showed that CP-CML CD34+ progenitors were characterized by significant lower phosphorylation of proteins involved in the regulation of growth and cell survival, such as tyrosine kinases of the Src family and members of STAT family, and by a significant higher phosphorylation of p53 (Ser15), compared to normal CD34+ cells from healthy donors. Consistent with these results, cell cycle analysis demonstrated that CP-CML CD34+ cells were characterized by higher percentage of cells in G0-phase compared to normal CD34+ cells. Analysis of expression profile on proteins involved in the apoptotic machinery revealed that, in addition, CD34+ cells from CP-CML were characterized by a significant lower expression of catalase and higher expression of HSP27 and FADD. In sum, we report that CD34+ cells from CP-CML are characterized by a proteomic and phospho-proteomic profile that promotes quiescence through the inhibition of proliferation and the promotion of survival. This differential signaling activation network may be addressed by novel targeted therapies aimed at eradicating CML stem cells.

## INTRODUCTION

Chronic myeloid leukemia (CML) is a clonal disorder originated from hematopoietic stem cells, pathogenically associated with reciprocal chromosomal translocation t(9;22), which gives rise to the fusion gene BCR-ABL, encoding for a protein of p210 kDa with a constitutively activated tyrosine kinase (TK) activity [1, 2]. Nowadays, the standard of care for patients with CML is represented by TK inhibitors (TKI activity) which inhibit the constitutive activation and subsequent proliferative functions of the onco-protein [3–8]. However, some patients become refractory to TKIs during treatment, suggesting the presence of resistance mechanisms. [9, 10]. While BCR-ABL mutations or amplification are generally recognized as the main causes of BCR-ABL-dependent mechanisms of TKI resistance, other studies have reported that the presence of quiescent Philadelphia positive (Ph+) stem cells could be an additional way to escape the TKIs activity [11–13].

Multiple mechanisms cooperate in promoting kinetic quiescence and prolonged survival of leukemic progenitor cells. However, many pathways involved in controlling these processes are also active in normal stem cells [14, 15]. Moreover, the activation status of specific signal transduction pathways (STP), such as PI3K/AKT/mTOR, Ras/Raf/MEK/ERK1/2, Wnt and STAT pathways [15, 16], as well as their interactions (cross-talking), play a crucial role both in normal and leukemic progenitor cell fate [17–20]. Revealing differentially activated components of these survival pathways could lead to a more effective eradication of the progenitor clonal cells, sparing normal counterparts.

While several studies have investigated at the genetic level the CML leukemia stem cells (LSCs) [15, 21–23], the post-translational modifications of the STP involved in the proliferation and survival machinery have not yet been explored.

The aim of this study was therefore to analyze at the protein level the expression profile and activation of numerous STP involved in the mechanisms of cell proliferation/quiescence and survival of CD34+ cells, obtained from samples of CML patients diagnosed in chronic phase (CP). Here, to the best of our knowledge, we report for the first time the proteomic and the phospho-proteomic profile of CD34+ cells obtained from CP-CML patients, compared to CD34+ progenitors from healthy donors, showing that the former are characterized by a specific proteomic and phospho-proteomic profile that promotes quiescence and survival.

## RESULTS

### Phospho-kinase proteomic profile of CD34+ cells from CP-CML patients

**Figure 1:**
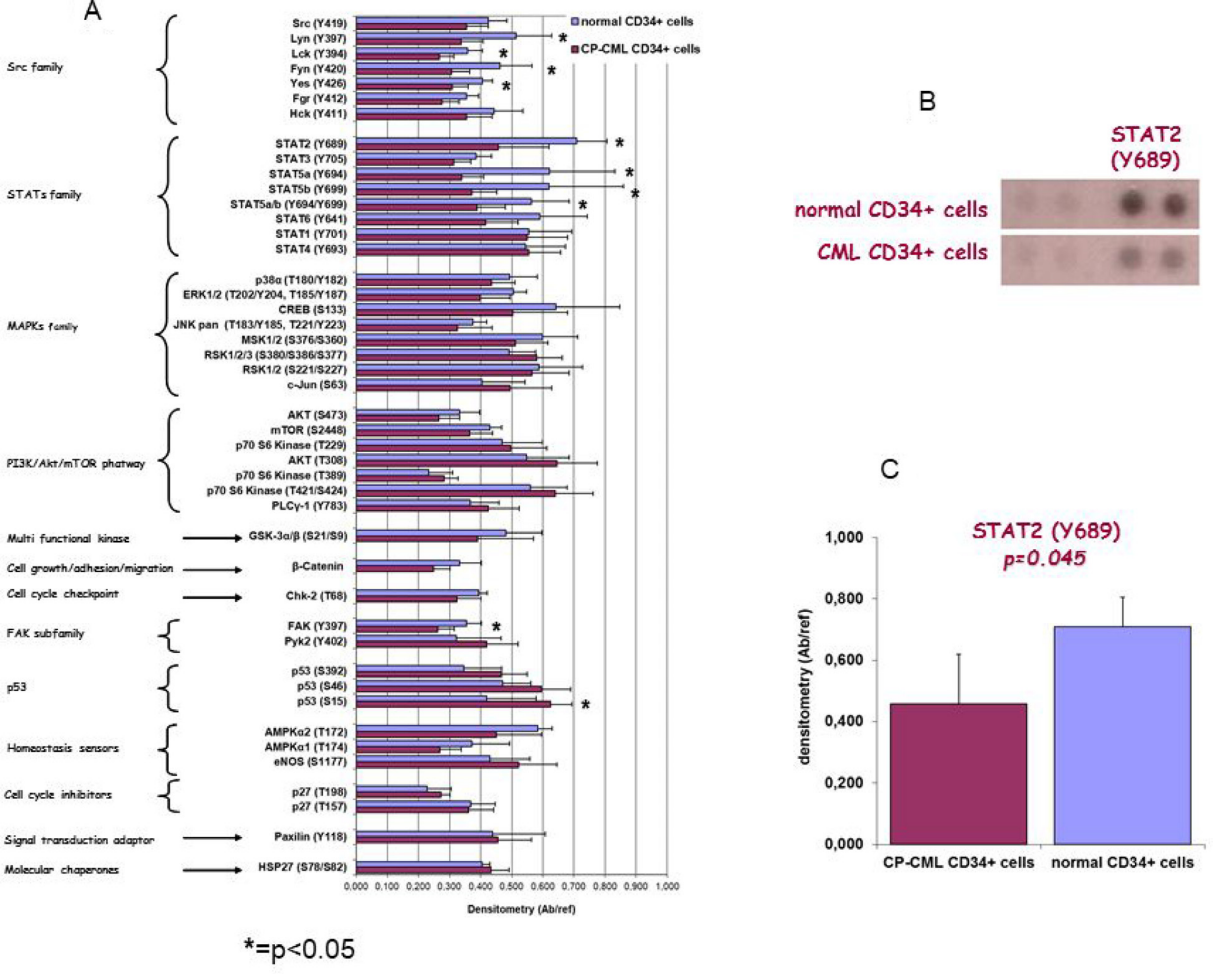
(**A**) Human phospho-kinase array kit was used to detect the relative levels of kinase phosphorylation in cell lysates from CP-CML CD34+ cells and from normal CD34+ cells. Bar diagram shows quantitation of the array data expressed as reported in Mat&Met section. (^*^p < 0.05 by two-tailed Student’s *t* test for the comparison between normal and CP-CML CD34+ cells). (**B**) Membrane hybridization and (**C**) densitometric quantitation are depicted for STAT2(Y689) as representative example.

Phospho-Kinase Array Kit was used to detect the activation status of 45 phosphorylation sites belonging to several STP in CP-CML CD34+ cells compared to normal CD34+ cells. Phospho-proteomic array analysis identified that several (a) non-receptor tyrosine kinases members of Src family, (b) components of different STP (p38, p-ERK1/2, CREB, MSK1/2, mTOR, GSK3a/b and β-catenin), and (c) key transcription factors including STAT family members were down-phosphorylated as compared to normal CD34+ cells (Figure 1). In particular, a statistically significant lower phosphorylation was found in four tyrosine kinases of the Src family (Lyn, Fyn, Yes: *p* = 0.02; Lck: *p* = 0.03), in three members of the STAT family (STAT2: *p* = 0.02; STAT5a and STAT5b: *p* = 0.045) and in FAK (*p* = 0.04). Moreover, an additional tyrosine kinase of the Src family (Fgr), two additional members of the STAT family (STAT3 and STAT6) and β-catenin resulted hypo-phosphorylated in CP-CML, although they did not reach statistical significance (between *p* = 0.06 and *p* = 0.1).

On the contrary, other signaling molecules as well as p53, p27, paxillin and HSP27 resulted hyper-phosphorylated in CP-CML CD34+ cells as compared to the normal CD34+ cells, but only p53 (Ser15) reached statistical significance (*p* = 0.047)

### Apoptotic proteomic profile of CD34+ cells from CP-CML patients

**Figure 2:**
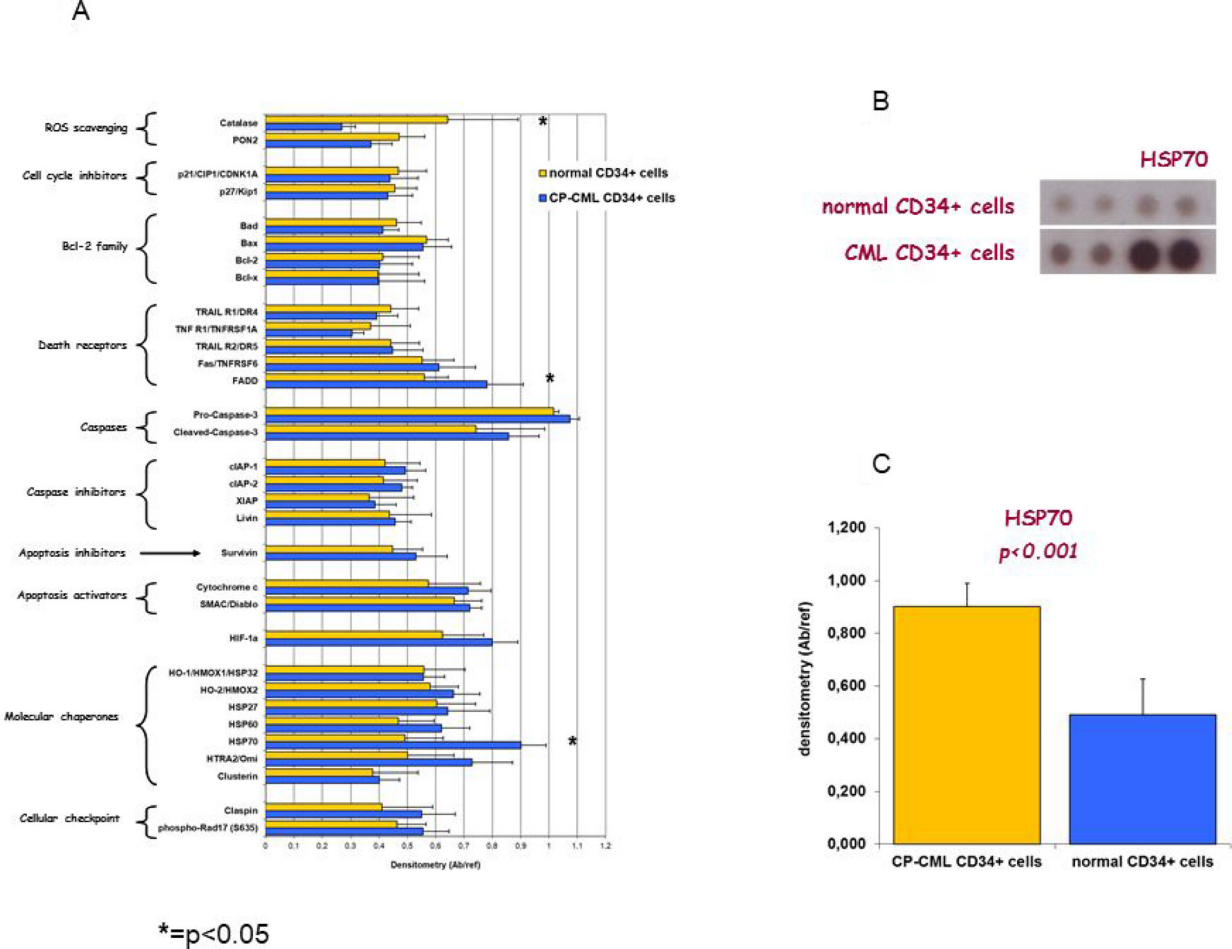
Cell lysates from CP-CML CD34+ cells and from normal CD34+ cells were analyzed for the expression of proteins which participate in apoptosis/cell survival modulation by using human apoptosis array kit (**A**) Bar diagram shows quantitation of the array data expressed as reported in Mat&Met section. (^*^p < 0.05 by two-tailed Student’s *t* test for the comparison between normal and CP-CML CD34+ cells). (**B**) Membrane hybridization and (**C**) densitometric quantitation are depicted for HSP70, as representative example.

Apoptosis Array Kit was used to analyze the expression of 32 apoptosis/cell cycle related proteins in CP-CML CD34+ cells compared to normal CD34+ cells. Results revealed that CD34+ cells from CP-CML, as compared to normal CD34+ cells, are characterized by: 1) lower expression of catalase (*p* = 0.012), an enzyme that protects cells from the toxic effects of hydrogen peroxide and promotes growth of normal and neoplastic cells including myeloid leukemia cells; 2) higher expression of FADD (*p* = 0.038), a death receptor involved in extrinsic apoptosis and necroptosis, and of HSP70 (*p* < 0.001), crucial for cells survival after toxic stimuli (Figure 2).

### Cell-cycle analysis and cell-cycle regulators’ protein expression

**Figure 3:**
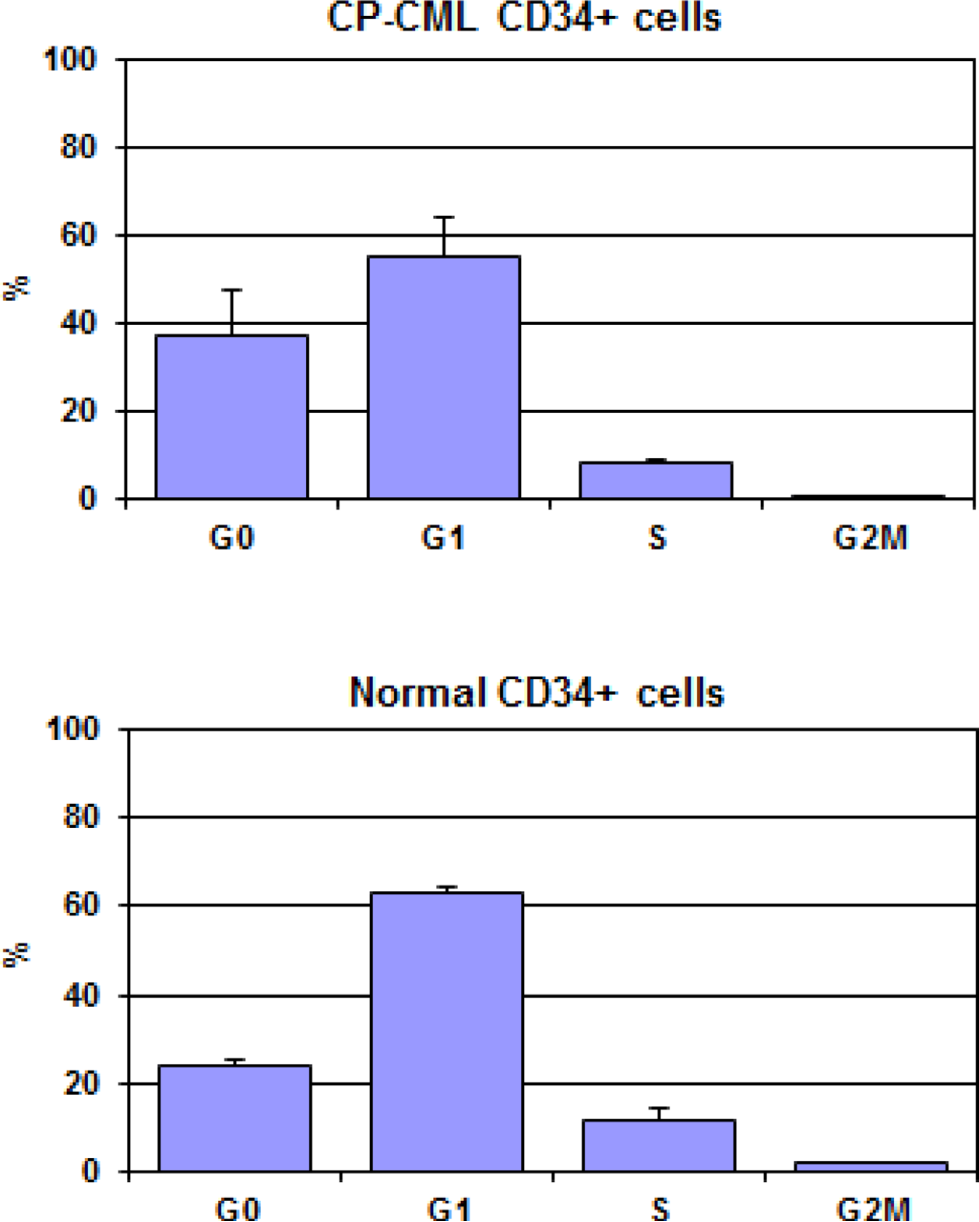
CP-CML CD34+ cells and normal CD34+ cells were analyzed by flow cytometry for cell cycle distribution as described in Mat&Met The results are expressed as mean percentage ± SD of cells in each phase of cell cycle.

We also performed the cell cycle analysis of the CD34+ cells from the aforementioned sources by the AO flow cytometric technique. The mean results are shown in Figure 3, demonstrating a lower percentage of cells in S-phase associated with a parallel increase of cells in G0-phase in CD34+ cells from CP-CML with respect to normal CD34+ cells indicating a larger pool of quiescent cells in CP-CML.

## DISCUSSION

CML is a stem cell disease sustained by a rare population of kinetic quiescent cells. In the indolent CP of the disease those dormant cells provide a reservoir for the Philadelphia chromosome. During the natural history of CML progenitors characterized by accumulated mutations due to increased genetic instability drive disease progression. As reviewed by Savona & Talpaz [15], in advanced phases, determinants of cell proliferation are indeed no longer addicted to BCR-ABL. Other aberrant signals induced by additional altered genes begin to autonomously control cell survival and proliferation, such as Src family kinase activation [15]. These observations could help explain why TKI therapy controls but fails to eliminate the disease.

Thus, one of the main goals for the eradication of leukemia should be to determine and to hit the sanctuary of quiescent CP-stem cells. Recent studies have reported that signaling pathways involved in the regulation of hematopoietic stem cells self-renewal and development are also active in cancer, thus representing the main open question on the differential expression of quiescent CP-CML progenitors from their normal counterpart [17, 24, 25].

Moreover, it was reported that the BCR-ABL oncogene and the resulting fusion protein activate multiple cross-talking STP, such as RAS/MEK/ERK, PI3K/Akt, Wnt, STAT5 and Src family kinases, potentially contributing to abnormal proliferation of clonal cells [17, 22, 25–27].

Based on this rationale, the aim of this study was to analyze, at the protein level, the expression profile and activation of numerous STP involved in the mechanisms of cell proliferation and survival of normal and CML CD34+, in order to identify differential molecular characteristics of CML progenitors, potentially druggable for leukemia eradication.

Previous studies have profiled the transcriptome of CML progenitors by gene array technology [21, 28–30]. Bruns and colleagues [21] have evaluated the transcriptional characteristics of CP-CML progenitors and of normal myeloid progenitor cells. They demonstrated that CP-CML progenitors have a transcriptionally more mature phenotype than its normal counterpart, showing downregulation of genes encoding adhesion molecules as well as decreased expression of transcription factors, stem-cell regulators and inhibitors of cell proliferation [21].

Information derived from genomic approaches have recently been also complemented and extended to the evaluation of proteomic profiles, which are able to highlight the protein post-translational modifications that play a major role in regulating protein activities, crosstalk and subcellular localization. In a pioneering study designed to identify cellular pathways affected by imatinib treatment, Xiong and colleagues [31] employed LC-MS/MS, through a SILAC approach, to assess and quantify the imatinib-induced protein expression variation in the BCR-ABL-positive human K562 cell line. Their results showed that imatinib induced a significantly altered level of expression in 73 out of 1344 quantified proteins [31]. Subsequently, the Kornblau group evaluated by Reverse Phase Protein Array (RPPA), the protein expression patterns of samples obtained from CML patients in order to differentially identify STP that predicted disease progression [32]. Although they used unsorted CML samples, Kornblau and colleagues reported that several proteins (HSP90, Rb, AIF, PP2A, Bcl-2, Xiap, Smad1, SSBP2a, PARP, Gab2, and TRIM24) were overexpressed in advanced-phase CML patients, as compared with those in CP [32]. Analysis of altered proteins related to blast crisis (BC), performed by using two-dimensional (2D) gel electrophoresis technique, identified 13 up-regulated proteins in BC-CML patients as compared to CP-CML, most of which were involved in the proteosome and in the small G-protein pathway [33]. Other studies have focused on the proteomic profile of CML. Peng and colleagues have evaluated the leukemia cell line K562 and their adriamycin-resistant counterpart [34], while Alaiya *et al.* have focused on the detection of protein biomarkers present in plasma from CML patients [35], showing the importance of this approach, which allowed the identification of both mechanism of adriamycin resistance and of molecular response to therapy, respectively.

Nevertheless, little is known about the proteomic profile of CML CD34+ progenitors. Thus, to the best of our knowledge, we analyzed for the first time the proteomic and the phospho-proteomic profile of CD34+ cells obtained from CP-CML, as compared to their normal CD34+ counterpart.

Phospho-proteomic array analysis between normal and CP-CML CD34+ cells revealed differences in the activation profile of several proteins. In particular, statistically significant differences were observed in members of the Src and of the STAT family (both involved in the regulation of growth and cell survival), in FAK, (a crucial regulator of cell migration) and in p53.

Our finding about the lower phosphorylation of four kinases belonging to Src family (Lck, Lyn, Fyn, Yes) in the CP of the disease is intriguing, since previous observations reported increased expression and phosphorylation of members of Src family in patients with CML in BC, evoking its role as possible mechanisms of resistance to TKI, independent from BCR-ABL [15, 36–38]. Specifically, Ban and co-workers have reported that the expression of Fyn is significantly increased in CML patients in BC as compared to those in CP and that the overexpression of BCR-ABL was associated with up-regulation of transcription and translation of Fyn [36]. Similarly, it has been shown that the overexpression of Lyn characterizes a more advanced stage of the disease [37]. Moreover, it was reported that the interplay between Syk and Lyn has a role in mediating nilotinib resistance in K562 cell line and in CD34+ cells isolated from CML patient blood samples [38].

Data from our group has previously demonstrated that progenitor biology largely depends on their kinetic status [29, 39]. Thus, we posed the question whether the differences and the similarities observed in the proteomic profile of CD34+ cells belonging to CP-CML compared to normal would reflect in the cell cycle distribution. To test this hypothesis, we performed a cell cycle analysis of the CD34+ cells by the AO flow cytometric technique demonstrating a larger pool of quiescent (dormant) cells in CD34+ cells from CP-CML with respect to normal ones. This result is in line with the lower activation of proteins belonging to STP mostly involved in cell proliferation (elements of Raf/MEK/ERK1/2 pathway, of Src or STATs family) of CD34+ cells from CP-CML patients.

**Figure 4:**
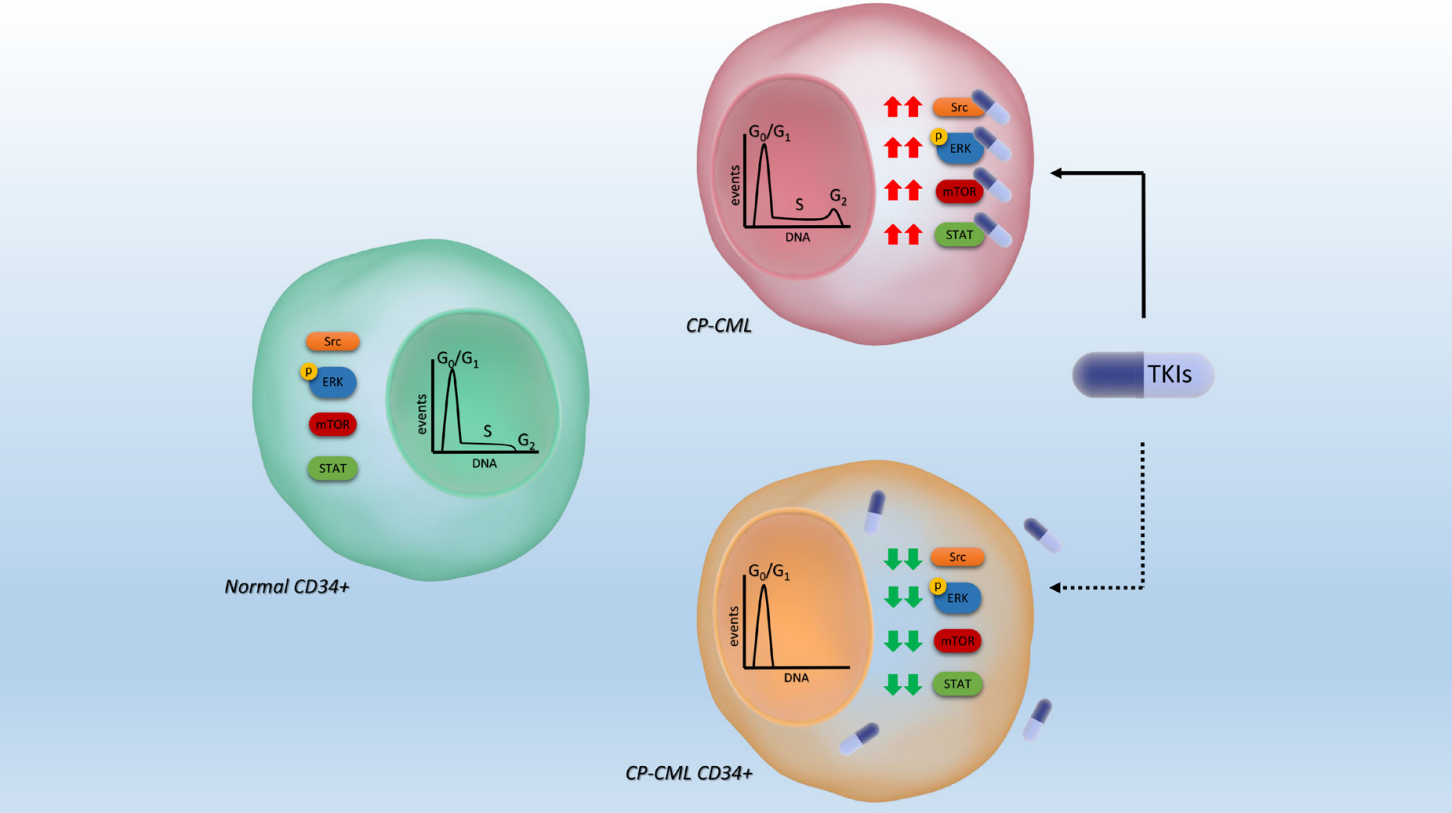
Schematic hypothetical model of CP-CML CD34+ cell biological characteristics as compared to normal progenitor and proliferating CML cells The downregulation of signals may confer TKI insensitiveness on CP-CML CD34+ progenitors. In the nuclei, cell cycle distributions are summarized.

Diaz-Blanco *et al.* [22], examining the genome-wide gene expression signature of highly enriched CD34+ cells from CP-CML patient, found that CP-CML CD34+ cells compared to normal CD34+ cells were characterized by a higher expression level of proliferation-associated genes (components of Raf/MEK/ERK1/2 and PI3K/AKT/mTOR pathway and some genes of the JNK and p38 MAPK pathways) [22]. In contrast, looking at a post-translational level, our results reveal a hypo-phosphorylation of the same proliferative pathways analyzed by gene profile in CP-CML CD34+ cells compared to normal CD34+. Our finding of an under-activation status of CP-CML CD34+ cells signaling is coherent with a low rate of cycling cells and with a reported resistance to cytotoxic cell cycle-related agents. The hypothetical model suggested by our results indicates that the quiescent CP-CML are characterized by a downregulation of the Src family, making them insensitive to TKIs. In the non-quiescent bulky population, the hyper-activation of the Src family members, as well as others STPs, provide the target for these agents. In this view, the TKIs resistance of CP-CML CD34+ is mainly due to lack of target, rather than to intrinsic mechanisms such as mutations or amplifications (Figure 4).

Moreover, we observed a significantly higher phosphorylation of p53 at Ser15 in CP-CML CD34+ cells compared to the normal CD34+ cells. Since it was demonstrated that phosphorylation of p53 on Ser15 by ATM, ATR, DNA-PK and by Chk2 upon DNA damage is crucial for inducing cell cycle arrest, our finding further supports the more pronounced resting status of CP-CML CD34+ cells. The role of p53 in CML has already been investigated by other groups. Carter and colleagues [40] have analyzed the expression of p53 and MDM2 in BC-CML cells and in proliferating and quiescent CD34+ CML progenitor cells, demonstrating a heterogeneous expression in cells from BC-CML patient samples. In addition they demonstrated that activation of p53 via MDM2 inhibition triggers apoptosis in blast cells, as well as in proliferating and quiescent CD34+ cells obtained from patients with BC-CML [40]. More recently, using an unbiased system biology approach, Abraham *et al.* [41] demonstrated that p53 and c-Myc are the key proteins in regulating the intracellular network of CML. They found that CML cells are characterized by higher c-Myc and lower p53 levels compared to normal CD34+ obtained from leukapheresis products or cord blood [41]. Moreover, they demonstrated that the simultaneous activation of p53 and inhibition of c-Myc decrease viability and induce apoptosis of CML LSCs without measurable effects on normal HSCs [41]. However, this elegant work does not include p53 post-translational modifications analysis. We investigated the p53 phosphorylation status at Ser15 also in CD34+ from cord blood and from leukapheresis product, observing a similar phosphorylation levels when compared to CD34+ cells obtained from CP-CML (data not shown), thus suggesting a possible role of the endosteal niche in controlling p53 phosphorylation.

More recently, our group has investigated the miRNA expression profile of different subpopulations of CML LSCs which have been previously shown to be endowed with TKI intrinsic resistance [42]. This study detected in the progenitor population from CP-CML the up-regulation of miR-29a-3p and miR-15 660-5p and the down-regulation of miR-494-3p, leading to a reduced susceptibility to the TKI-induced apoptosis. These results demonstrated that aberrant miRNA expression in CML could contribute to the intrinsic TKI-resistance via proliferative and apoptotic aberrant RNA-regulation.

In conclusion, we report here that CD34+ progenitor cells obtained from CP-CML patients, compared to CD34+ from healthy donors, are characterized by a specific proteomic and phospho-proteomic profile that promotes quiescence and survival.

These observations strongly make the case for the necessity of a deeper understanding of molecular mechanisms that are deregulated in the progenitors of CP-CML as compared to normal counterpart. The identification of differentially activated STP crucial for CML progenitor cell survival, resistance and progression should therefore lead to novel and more efficient drug design and therapies aiming to eradicate and not only to control the disease. In this respect, potentially new druggable targets have recently been identified: from survival factor belonging to different STP (such as Bcl6, GSK3β, BLK, CD25, CD70, SIRT1 SMO, etc.) to p53, c-Myc, HDAC and to metabolic enzyme (i.e. ALOX5) [16]. Accordingly, several compounds able to modulate those targets have been evaluated in clinical trials. Further pre-clinical evaluation of the role of these new molecular targets, together with innovative technologies, will finally enable to develop innovative therapeutic approach based on quiescent CML progenitor eradication.

## MATERIALS AND METHODS

### CD34+ cells

Ph+ CD34 + cells were purified by immunomagnetic separation from peripheral blood (PB) of seven patients with newly diagnosed CML patients in chronic phase, with a white blood cell count ranging between 41,900 and 421,400. Six out of seven were with Intermediate Sokal Score, one with Low Score. The BCR-ABL fusion transcript was e14a2 (b3a2) in five patients, e13a2 (b2a2) in one patient; the coexistence of e14a2 and e13a2 was observed in the remaining case. Normal CD34+ cells, obtained from four normal bone marrow of healthy donors were purchased from StemCell Technologies (Vancouver Canada). CML samples were obtained from the Hematology Unit, Policlinico Sant’Orsola-Malpighi, Bologna, Italy. Written informed consent for *in vitro* studies was obtained from all patients in accordance with the Declaration of Helsinki. Cell number and viability were determined by triplicate trypan blue exclusion.

### Protein expression profile

Protein extracts were obtained by treating the cell samples for 30 minutes in ice with a lysing buffer containing 10 mM NaF, 1 mM Na3VO4, 150 mM NaCl, 1 mM MgCl2, 1 mM CaCl2, 0.1% NaN3, 10 mM iodoacetamide, 3 mM PMSF, 1% Triton-X100 supplemented with a cocktail of protease inhibitors (Roche Diagnostic Corp., Indianapolis, IN). Cell lysates were then centrifuged at 13000 rpm for 5 minutes and the protein material suspended in the supernatant was collected and quantified by spectrophotometer. The activation state of 45 phosphorylation sites belonging to several intracellular STP kinases (Table 1) and the expression of 32 proteins most directly involved in the apoptotic machinery (Table 2) were quantified in 6/7 and in 5/7 samples, respectively, by using an antibody direct phase array (Human Phospho-Kinase Array Kit, ARY003, and Human Apoptosis Array Kit, ARY009 respectively; Proteome Profiler™ R&D Systems, Abingdon, UK). The assays were performed in accordance with the manufacturer’s instructions. Briefly, cell lysates were diluted, layered on nitrocellulose membranes and incubated overnight at 4° C on a rocking platform. The membranes were washed to remove unbound proteins and incubated with a cocktail of biotinylated detection antibodies for 2 h. The subsequent application of Streptavidin-HRP and chemiluminescent detection reagents (ECL) allowed displaying a signal for each point of capture, corresponding to the amount of bound protein. Multiple time exposures were used to verify the linearity of the samples analyzed. The spots obtained were scanned and quantified by densitometric analysis using ImageJ software (NIH) and results were expressed as arbitrary units (a.u.).

**Table 1:** List of proteins and corresponding phosphorylation site detected in the study

Phospho-proteins	
MAPKs family	p38α (T180/Y182)
	ERK1/2 (T202/Y204, T185/Y187)
	CREB (S133)
	JNK pan (T183/Y185, T221/Y223)
	MSK1/2 (S376/S360)
	RSK1/2/3 (S380/S386/S377)
	RSK1/2 (S221/S227)
	c-JUN (S63)
PI3K/Akt/mTOR pathway	AKT (S473)
	AKT (T308)
	mTOR (S2448)
	p70 S6 Kinase (T389)
	p70 S6 Kinase (T421/S424)
	p70 S6 Kinase (T229)
	PLCγ-1 (Y783)
STATs family	STAT2 (Y689)
	STAT3 (Y705)
	STAT5a (Y694)
	STAT5b (Y699)
	STAT5a7b (Y694/Y699)
	STAT6 (Y641)
	STAT1 (Y701)
	STAT4 (Y693)
p53	p53 (S392)
	p53 (S46)
	p53 (S15)
Src family	Src (Y419)
	Lyn (Y397)
	Lck (Y394)
	Fyn (Y420)
	Yes (Y426)
	Fgr (Y412)
	Hck (Y411)
FAK subfamily	FAK (Y397)
	Pyk2 (Y402)
Multi functional kinase	GSK-3α/β (s21/S9)
Cell cycle checkpoint	Chk-2 (T68)
Cell cycle inhibitors	p27 (T198)
	p27 (T157)
Cell growth,adhesion and migration	β-Catenin
Molecular chaperones	HSP27 (S78/S82)
Homeostasis sensors	AMPKα1 (T174)
	AMPKα2 (T172)
	eNOS (S1177)
Signal transduction adaptor	Paxillin (Y118)

**Table 2:** List of apoptosis related proteins detected in the study

Apoptosis related proteins	
**Bcl-2 family**	Bad
	Bax
	Bcl-2
	Bcl-x
**Death receptors**	TRAIL R1/DR4
	TRAIL R2/DR5
	FADD
	Fas/TNFRSF6
	TNF R1/TNFRSF1A
**Caspase**	Pro-Caspase-3
	Cleaved-Caspase-3
**Apoptosis inhibitors**	cIAP-1
	cIAP-2
	XIAP
	Livin
	Survivin
**Apoptosis activators**	Cytochrome c
	SMAC/Diablo
**Molecular chaperones**	HO-1/HMOX1/HSP32
	HO-2/HMOX2
	HSP27
	HSP60
	HSP70
	HTRA2/Omi
	Clusterin
**ROS scavenging**	Catalase
	PON2
**Cell cycle inhibitors**	p21/CIP1/CDNK1A
	p27/Kip1
**Cellular checkpoint**	Claspin
	phospho-Rad17 (S635)
**Transcription factor**	HIF-1a

The average signal of the pair of duplicate spots, representing each protein, was calculated after subtraction of background values (pixel density) from negative control spots and normalization to average values from internal positive control spots.

### Cell cycle analysis

Cell cycle distribution changes were evaluated using the Acridine Orange (AO) technique described previously [43]. The percentage of cells in the G0, G1, S, and G2M were determined by measuring simultaneously the DNA and RNA total cellular content. The percentage of apoptotic cells was measured based on the decreased stainability of apoptotic elements in DNA green fluorescence (sub-G1 peak on DNA-frequency histograms) coupled with a higher RNA red fluorescence (which is common to chromatin condensation); cell debris were excluded from the analysis on the basis of their forward light scatter properties. Cell-cycle distribution was analyzed using the ModFit LT software (Verity Software House, Topsham, ME).

### Statistical analysis

Statistical analysis was performed using Microsoft Excel software. Student’s *t* test was used to analyze significant differences. Results were considered to be statistically significant if *P* < 0.05. Results were expressed as mean ± SE between groups.
